# Effects of a 12-Week Web-Based Weight Loss Program for Adults With Overweight and Obesity on COVIDAge and Lifestyle-Related Cardiometabolic Risk Factors: A Randomized Controlled Trial

**DOI:** 10.3389/fpubh.2022.868255

**Published:** 2022-05-20

**Authors:** Judith Brame, Christoph Centner, Niklas Berg, Matt Bartlam, Albert Gollhofer, Daniel König

**Affiliations:** ^1^Department of Sport and Sport Science, University of Freiburg, Freiburg, Germany; ^2^Everist Health, Ann Arbor, MI, United States; ^3^Department of Sport Science, Institute for Nutrition, Sports and Health, University of Vienna, Vienna, Austria; ^4^Department of Nutritional Sciences, Institute for Nutrition, Sports and Health, University of Vienna, Vienna, Austria

**Keywords:** COVID-19, lifestyle, overweight, obesity, weight loss, web-based program, cardiometabolic risk factors

## Abstract

The coronavirus disease 2019 (COVID-19) pandemic has induced unhealthy lifestyles, particularly an increase in overweight and obesity, which have been shown to be associated with an increased risk of unfavorable COVID-19 outcomes. Web-based health programs could be a helpful measure, especially in times of severe restrictions. Therefore, the present study aimed to investigate the effects of regular attendance in a 12-week web-based weight loss program on COVIDAge, a new construct for risk assessment of COVID-19, and lifestyle-related cardiometabolic risk factors. *N* = 92 subjects with overweight and obesity (50.0 ± 10.8 years, 76.1% females, 30.5 ± 2.1 kg/m^2^) of this randomized controlled trial, which were assigned to an interactive (ONLINE: intervention group) or non-interactive (CON: control group) web-based weight loss program, were included in the data analysis. COVIDAge and cardiometabolic risk factors, including anthropometric outcomes, blood pressure, flow-mediated dilatation, and blood parameters, were assessed before and after the 12-week intervention phase. There was a significant group difference in the change of COVIDAge (ONLINE: −4.2%, CON: −1.3%, *p* = 0.037). The ONLINE group also showed significantly greater reductions in anthropometric outcomes and systolic blood pressure than the CON group (*p* < 0.05). To the authors' knowledge, this was the first study investigating the effects of regular attendance in a web-based health program on lifestyle-related risk factors for COVID-19. The results demonstrated that adults with overweight and obesity can improve their COVIDAge and specific cardiometabolic risk factors by using this interactive web-based weight loss program regularly. However, this needs to be confirmed by future studies. This study is registered at the German Clinical Trials Register (DRKS00020249, https://www.drks.de).

## Introduction

In late 2019, the first coronavirus disease 2019 (COVID-19) cases caused by severe acute respiratory syndrome coronavirus type 2 were discovered in China (1). After more than 2 years, the World Health Organization reported more than 364 million confirmed cases and more than 5.6 million deaths worldwide related to COVID-19 (2). Due to the exponentially increasing number of infections, governments implemented drastic measures such as travel restrictions, quarantine, isolation, social distancing, and face masks to reduce the total number of human interactions (3, 4).

Consequently, dietary and physical activity behavior could change in health-compromising ways during phases of quarantine, isolation, or lockdown (5). Recent evidence suggests that dietary habits have worsened during COVID-19 home confinement, as indicated by increased consumption of highly processed food, an increased number of snacks between meals, and an overall increase in meals per day (5). This is noteworthy as short-term overfeeding over only 1 week may already lead to a significant increase in body weight (6). In addition, even short-term reductions in physical activity over 14 days may result in metabolic dysfunction, increased intraabdominal fat tissue, and hyperinsulinemia (7, 8). So, these lifestyle behaviors could further increase the prevalence of overweight and obesity in almost every country in the world (9, 10) and are known to be related to major cardiometabolic risk factors such as hypertension, dyslipoproteinemia, and cardiovascular disease (11, 12). For COVID-19, obesity is associated with a notably increased risk of unfavorable outcomes (13). Moreover, type 2 diabetes mellitus, strongly associated with overweight and obesity, also seems to be a critical comorbidity in terms of severity in patients with COVID-19 (14).

To counteract weight gain and increased cardiometabolic risk, besides the well-known advice to maintain a healthy lifestyle, people should consider participating in web-based weight loss programs (15). Especially in times of distance rules and reduced interpersonal contacts, these programs could provide helpful support to counteract the adverse effects of COVID-19 restrictions (16) and thus the increased risk of adverse COVID-19 outcomes (3, 7, 9, 17).

To investigate the influence of lifestyle-related risk factors for COVID-19, a construct accounting for the heterogeneity of risk factors is needed. The COVIDAge Risk Calculator™ (18) represents a first step toward calculating such a construct by integrating lifestyle-related risk factors for COVID-19. It combines chronological age and different lifestyle factors associated with an increased risk for COVID-19 through an algorithm based on the latest research in the field (19). The calculated outcome results in the so-called “COVIDAge”. The present study aimed to examine the effects of regular attendance in a 12-week web-based weight loss program for adults with overweight and obesity on COVIDAge and lifestyle-related cardiometabolic risk factors.

## Materials and Methods

### Study Design

The study was designed as a randomized controlled, parallel-group clinical trial as part of a nationwide online trial evaluating a 12-week web-based weight loss program (20). In order to examine physiological health outcomes, all participants of the online trial living in Southwest Germany were invited to medical examinations in addition to online questionnaires. These examinations were carried out before and after the 12-week intervention phase at the Department of Sport and Sport Science of the University of Freiburg between January and August 2020. The study was reviewed by the Ethics Committee of the University of Freiburg (237/19), following the principles of the Declaration of Helsinki, and registered at the World Health Organization approved German Clinical Trials Register (DRKS00020249, https://www.drks.de). Detailed study information can be found in the published study protocol (21).

### Participants

Healthy women and men were included in the trial if they were aged between 18 and 65 years and had a body mass index (BMI) ranging from 27.5 to 34.9 kg/m^2^. In case of known health problems or diseases, a medical certificate from the general practitioner to participate in the trial was required. Exclusion criteria were defined as being pregnant or breastfeeding. Recruitment methods comprised print and online media, especially the local press, radio and flyers, google advertisements, and recruitment activities of the involved institutions (websites, newsletters, social media) (21).

All recruitment efforts focused on directing interested subjects to a landing page to inform and register for the study, including a written informed consent form. Subsequently, participants were screened by telephone regarding the inclusion and exclusion criteria. After successful screening, an appointment was fixed for the first medical examination. Seven days after this examination, participants were given access to a 12-week web-based weight loss program. The second medical examination was realized after program completion (21).

### Interventions

#### Interactive Web-Based Weight Loss Program

The program delivered to the intervention group (ONLINE) was one of the coaching modules (“TK-WeightLossCoaching”) of a multimodal health program (“TK-HealthCoach”) offered by a national statutory health insurance fund (Techniker Krankenkasse). With an interactive, self-directed, and flexible usability, this program enables personalized health coaching according to the users' health profiles (22). The primary goal of the weight loss coaching module is to lose and maintain weight within a 12-week intervention phase based on the energy density concept (23) and a balanced, healthy diet to prevent chronic degenerative diseases (24).

For program setup, users first completed a multi-step anamnesis and set their personal weight loss goal (3 or 5 kg). They were then taken to an online platform made up of three areas. The first area (“My health program”) displayed a personal dashboard as a weekly and an overall 12-week coaching plan [phase 1: getting to know and trying out (week 1–3), phase 2: consolidating new behaviors (week 4–6), phase 3: anchoring habits (week 7–12)] that users compiled and modified according to their individual needs. Thereby they could choose from a wide range of mainly 12 weight loss activities, such as “I achieve a green energy density” or “I eat fruit two times and vegetables three times a day,” to be supported in implementing the energy density concept and the principles of a balanced, healthy diet. One preset daily activity was the nutritional protocol, as it was the main activity of the weight loss program. It provided a helpful management tool on energy density, calorie balance, and fluid intake and included an analysis of macronutrients and lots of healthy recipes. Another preset weekly activity was to report body weight and waist circumference. All completed activities had to be logged daily or weekly. Moreover, the dashboard featured current health goal achievements (weight and waist circumference loss), daily tips, notifications, and support opportunities. The second area (“Knowledge”) supplied scientific background information on different health topics (e.g., diet, physical activity, stress management) through interactive knowledge courses with texts, videos, tools, and tests. One course (“Weight loss with energy density concept”) was an integral part of the coaching plan to teach users the energy density concept. Finally, in the third area (“My data”), users could view an analysis profile of logged activity data as well as their personal and health profile data, study information including written informed consent, and the option to withdraw study participation. To monitor physical activity behavior, users received an activity tracker (Fitbit Charge 3™) and could synchronize it with the program. At the end of the program, users obtained a coaching summary of their achieved successes (21).

The program was provided as a web-based version that could be used on various electronic devices, such as computers, laptops, tablets, or smartphones. This made the program easily accessible to users anywhere and at any time. Furthermore, the users could adapt the program flexibly to their individual needs, which kept the program interesting and varied. In addition, the program tried to accompany the users in everyday life by providing daily activities, tips, and notifications. All these factors together should increase the acceptance and success of the program. On the other hand, there were some potential barriers to program acceptance and success. Because the program was designed on a scientific, evidence-based foundation, the program usability could be perceived as quite complex. Consequently, program use could be considered time-consuming. This could be very challenging, especially in times of the COVID-19 pandemic in a stressful private and professional situation.

#### Non-interactive Web-Based Weight Loss Program

The control group (CON) program was similarly designed as an online platform to assist users in losing and maintaining weight over a 12-week intervention phase. However, this program merely offered a health-related knowledge transfer. Therefore, the first area (“My health program”) presented only evidence-based information on various health topics, particularly on the energy density concept and a healthy diet. These contents were taught in small lessons and without any interactive components. In the second area (“My data”), users got personal data, study information, including written informed consent, and the option of withdrawing study participation. For monitoring physical activity behavior, participants of the control group also received an activity tracker (Fitbit Charge 3™) but could not link it to the program. After study completion, access to the interactive web-based weight loss program was provided (21).

### Outcomes

#### Primary Outcome

The primary outcome (COVIDAge) comprised a construct newly developed during the COVID-19 pandemic by Everist Health to estimate an individual's risk for complications of COVID-19 infection (25). It covers a combination of chronological age and various lifestyle factors associated with COVID-19 outcomes (19). Therefore, a higher COVIDAge indicates a higher risk of COVID-19 (18). In the present study, the calculation of COVIDAge in days was accomplished using the COVIDAge Risk Calculator™ (Version 1.0.0) (https://calculator.covid-age.com) (18), considering several lifestyle factors measured within the medical examinations.

#### Secondary Outcomes

These lifestyle factors were defined as secondary outcomes. First, anthropometric outcomes, including body height, body weight, BMI, and waist circumference, were assessed by stadiometer (seca 274), bioelectrical impedance analysis scale (seca mBCA 515), and measuring tape (seca 201) (26). Flow-mediated dilatation and systolic and diastolic blood pressure were measured using the non-invasive AngioDefender™ system (27). Blood glucose (HbA_1c_) and blood lipid (triglycerides, HDL cholesterol, LDL cholesterol) levels were taken from blood samples. Furthermore, diseases, medication, and daily cigarette consumption were asked *via* anamnesis. In order to perform these examinations in a standardized manner, participants appeared fastened (meal and drinks: ≥ 12 h, alcohol consumption and exercise: ≥ 48 h), and all examinations were preferably performed at the same time of the day (21).

### Sample Size

The sample size calculation was done based on the initially defined primary outcome of the clinical trial (“body weight”) using the G^*^Power software (Version 3.1) (RRID: SCR_013726) (28). Assuming previously observed effect sizes from comparable studies (29, 30) as well as a two-sided significance level of 0.05 and a statistical power of 0.80, the required number of cases was estimated to be *n* = 64 for the intervention and control group, respectively. Considering an additional dropout rate of 15%, the total target sample size was calculated to be *n* = 75 for both the intervention and control group (20, 21).

### Randomization

With successful online registration, participants were immediately assigned to the intervention and control group by permuted block randomization with variable block sizes of 4, 6, and 8 and an allocation ratio of 1:1. The random allocation sequence was generated by the Section of Health Care Research and Rehabilitation Research of the Medical Center of the University of Freiburg using the RITA software (Version 1.50) (31). The sequence was then transmitted to Vilua Healthcare GmbH, where it was securely stored and finally computer-automated implemented according to the randomization lists. Permuted block randomization was chosen to avoid unequal distributions in the study groups if, intended and achieved, case numbers would differ (20, 21).

### Blinding

The study participants could not be blinded due to study information, i.e., the allocation to an interactive and non-interactive web-based weight loss program. In contrast, the clinical trial's healthcare providers and outcomes assessors could be blinded as they would know about group allocation only after data collection was completed. Nevertheless, it could not be ruled out that study participants might report allocation at the medical examinations. The data analysts also could not be blinded because group allocation might have been suspected on the data (20, 21).

### Statistical Methods

For all statistical analyses, the IBM SPSS Statistics software (Version 27.0.0.0) (RRID: SCR_019096) (32) was used. Normal distribution was confirmed by the Shapiro-Wilk test. In case of not meeting the normality assumption, the respective variables were log-transformed. Variance homogeneity was assessed using the Levene test. To investigate potential group differences regarding the primary outcome and anthropometric variables at baseline, independent *t*-tests were calculated to compare both groups. For secondary outcomes analyses, repeated measures analyses of variance were computed.

The level of significance was set at 0.05 for all comparisons. Participants of the intervention group with a program adherence of ≤ 50% (i.e., no or at least one logged activity per week in 1–6 weeks of the web-based weight loss program) were excluded from the analysis. This was done to examine program effectiveness based on regular attendance in the weight loss program. Thus, our aim was not to investigate the dose-response relationship within the weight loss program but to examine the effects of a regularly used weight loss program compared to a control intervention. The following data are presented as mean ± SD if not indicated otherwise.

## Results

### Study Participants' Characteristics

**Figure 1:**
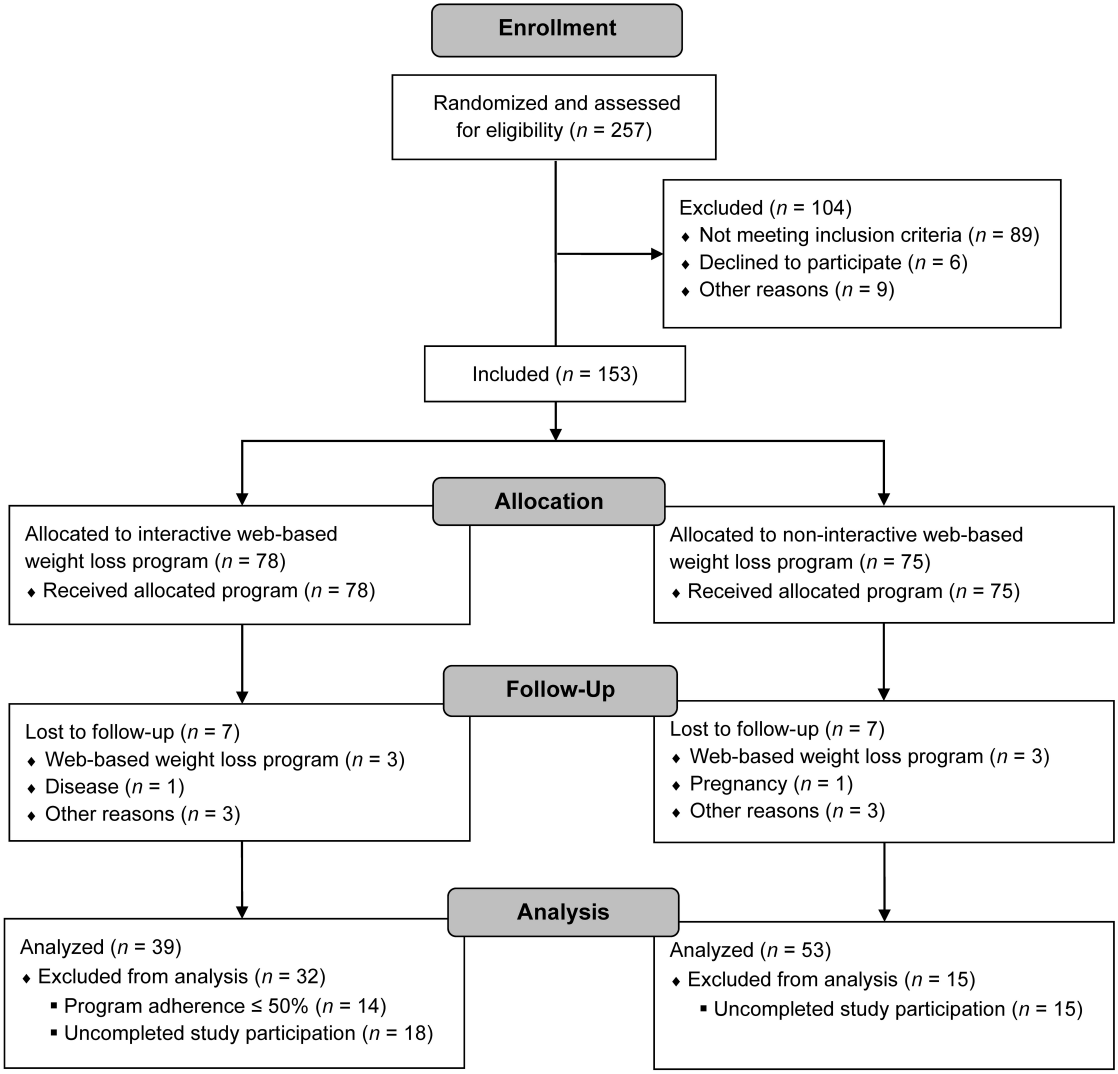
Study participant flow.

Study participants were recruited from January to July 2020. After randomization and successful screening, they attended the first medical examination and the second after completing the 12-week weight loss program. *N* = 53 of the intervention and control group, respectively, had already completed the second medical examination at the time of data analysis. In total, *n* = 7 dropouts were reported in each study group. None of the dropouts was associated with any side effects of the study. There were also no adverse events reported in either study group. For the following analyses, participants of the intervention group with an adherence rate of > 50% were included (*n* = 39). Details of the study participant flow are illustrated in Figure 1. Baseline characteristics of the study participants are shown in Table 1. At baseline, none of the anthropometric variables was significantly different between both study groups (*p* > 0.05).

**Table 1:** Study participants' characteristics (*n* = 92, ONLINE: *n* = 39, CON: *n* = 53).

**Variable**	**Group**	**MEAN ± SD**	**MIN**	**MAX**
Female, n (%)	TOTAL	70 (76.1)	-	-
	ONLINE	34 (87.2)	-	-
	CON	36 (67.9)	-	-
Age (years)	TOTAL	50.0 ± 10.8	23	65
	ONLINE	50.7 ± 11.0	23	65
	CON	49.6 ± 10.8	23	65
Height (cm)	TOTAL	169.0 ± 7.6	153.9	191.1
	ONLINE	167.9 ± 6.5	153.9	184.2
	CON	169.7 ± 8.3	156.4	191.1
Weight (kg)	TOTAL	87.2 ± 10.7	69.5	120.9
	ONLINE	85.6 ± 9.3	69.5	115.6
	CON	88.4 ± 11.6	70.2	120.9
BMI (kg/m^2^)	TOTAL	30.5 ± 2.1	27.5	34.9
	ONLINE	30.3 ± 2.2	27.8	34.9
	CON	30.6 ± 2.0	27.5	34.9

*ONLINE, intervention group; CON, control group; BMI, body mass index*.

### COVIDAge

**Figure 2:**
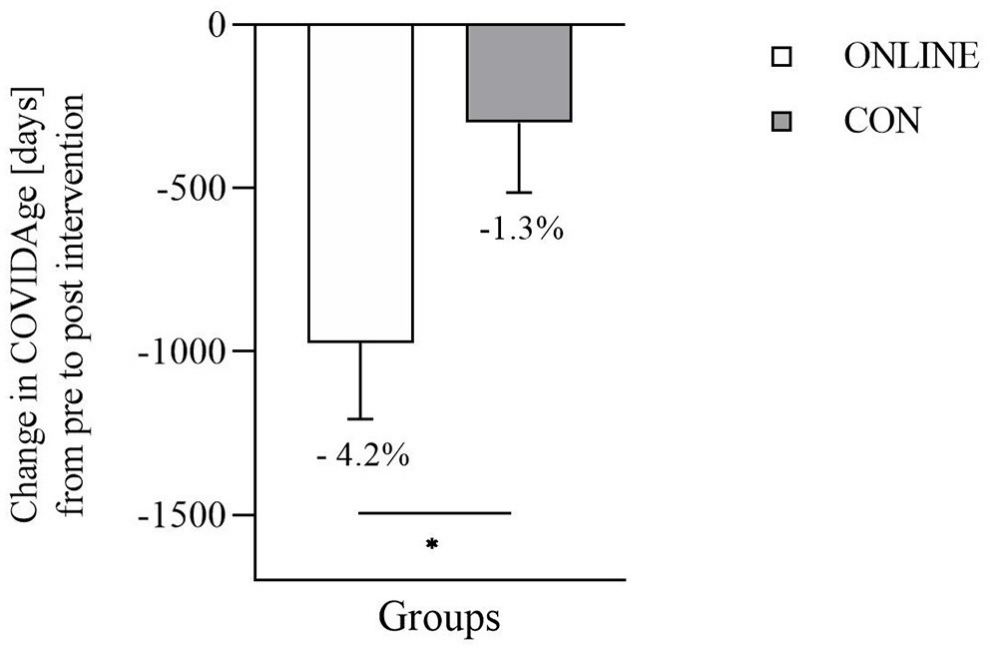
Change in COVIDAge (days) from pre to post intervention (*n* = 92, ONLINE: *n* = 39, CON: *n* = 53) (ONLINE, intervention group; CON, control group). Data are presented as mean ± SEM. * indicates a significant difference in independent *t*-test (*p* = 0.037, *d* = 0.446).

From pre to post intervention, the ONLINE group decreased COVIDAge by −4.2% from 23,152 ± 6,073 days (~ 63.4 years) to 22,178 ± 5,761 days (~ 60.7 years). In contrast, a decline of −1.3% in COVIDage was observed in the CON group [pre: 23,053 ± 7,409 days (~ 63.2 years), post: 22,754 ± 7,348 days (~ 62.3 years)]. There was a significant group difference (*p* = 0.037, *d* = 0.446; Figure 2).

### Lifestyle-Related Cardiometabolic Risk Factors

Participants in the ONLINE group demonstrated a significantly greater decrease in anthropometric outcomes than in the CON group [body weight (*p* = 0.000, $\eta_{p}^{2}$ = 0.168), BMI (*p* = 0.000, $\eta_{p}^{2}$ = 0.146), waist circumference (*p* = 0.005, $\eta_{p}^{2}$ = 0.087)]. Additionally, systolic blood pressure decreased significantly more in the ONLINE group than in the CON group (*p* = 0.046, $\eta_{p}^{2}$ = 0.043). No further significant interaction effects (time **×** group) were observed in any other lifestyle-related cardiometabolic risk factor (Table 2).

**Table 2:** Lifestyle-related cardiometabolic risk factors (*n* = 92, ONLINE: *n* = 39, CON: *n* = 53).

**Variable**	**Group**	**PRE**	**POST**	**rmANOVA (time × group)**	** $\eta_{\text{p}}^{2}$ **
Weight (kg)	ONLINE	85.6 ± 9.3	82.0 ± 9.7	0.000^*^	0.168
	CON	88.4 ± 11.6	87.2 ± 11.9		
BMI (kg/m^2^)	ONLINE	30.3 ± 2.2	29.1 ± 2.6	0.000^*^	0.146
	CON	30.6 ± 2.0	30.1 ± 2.2		
WC (cm)	ONLINE	100.9 ± 8.8	97.2 ± 9.5	0.005^*^	0.087
	CON	100.4 ± 9.4	98.8 ± 9.9		
Systolic BP (mmHg)	ONLINE	124.6 ± 12.3	118.2 ± 12.7	0.046^*^	0.043
	CON	121.2 ± 14.5	119.2 ± 14.6		
Diastolic BP (mmHg)	ONLINE	77.9 ± 9.3	74.2 ± 7.8	0.430	0.007
	CON	76.0 ± 9.4	73.5 ± 9.2		
FMD (%)	ONLINE	9.0 ± 1.7	9.0 ± 2.0	0.990	0.000
	CON	9.1 ± 1.7	9.1 ± 1.7		
HbA_1c_ (%)	ONLINE	5.5 ± 0.4	5.4 ± 0.4	0.333	0.010
	CON	5.4 ± 0.4	5.4 ± 0.4		
Triglycerides (mg/dl)	ONLINE	112.0 ± 43.5	103.7 ± 35.0	0.804	0.001
	CON	119.7 ± 54.9	120.0 ± 71.9		
HDL-C (mg/dl)	ONLINE	57.7 ± 10.7	57.1 ± 8.8	0.797	0.001
	CON	58.2 ± 12.8	57.1 ± 12.1		
LDL-C (mg/dl)	ONLINE	130.0 ± 25.2	128.5 ± 26.7	0.742	0.001
	CON	137.6 ± 37.8	134.2 ± 36.5		

*Data are presented as mean ± SD*.

* *indicates a significant interaction effect (time × group) in repeated measures analysis of variance (rmANOVA) (p < 0.05). ONLINE, intervention group; CON, control group; BMI, body mass index; WC, waist circumference; BP, blood pressure; FMD, flow-mediated dilatation; HDL-C, HDL cholesterol; LDL-C, LDL cholesterol*.

## Discussion

The findings from the present study indicated that a regularly used, interactive, web-based weight loss program significantly decreased COVIDAge as well as specific cardiometabolic risk factors (body weight, BMI, waist circumference, and systolic blood pressure) in adults with overweight and obesity.

The construct of COVIDAge has been designed to indicate COVID-19 severity and risk of associated complications. The scale incorporates all major determinants and relies on recent scientific data, including the 10 year Framingham study (33). This simplification approach aims to make this topic more accessible for the population at large and thus provide higher awareness around COVID-19. As a predictor variable of COVIDAge, several dimensions of overweight and obesity, including BMI and waist circumference, have been directly and indirectly associated with a higher risk of COVID-19 complications. On the one hand, a direct link has been demonstrated by Popkin and colleagues (9), who found that patients with obesity have an elevated risk of being positively tested for COVID-19 (+49%). This also resulted in an increased mortality risk of COVID-19 (+48%) (9). On the other hand, obesity also indirectly impacts the risk of COVID-19 complications by increasing cardiovascular events (34).

During the COVID-19 pandemic, the importance of home-based health programs significantly increased. Implementing non-pharmacological countermeasures, such as lockdowns or orders to stay and work at home, has essentially contributed to reducing the spreading and thus mortality of COVID-19 (35, 36). From the point of view of overall physical health, however, these interventions have also been disruptive since general health determinants such as physical inactivity (37) and mental stress (38) increased and thus may lead to the development of overweight and obesity (3).

Besides face-to-face interventions (39), previous research revealed that web-based intervention programs are an effective tool for facilitating weight loss and lifestyle changes (40). The present study demonstrated that a regularly used 12-week web-based health program facilitated weight loss of 4.2% in the intervention group and 1.4% in the control group. Similarly, BMI decreased by 4.0% and 1.6% in the intervention and control group, respectively. These changes are in accordance with previous trials, which demonstrated comparable results in either face-to-face (41) or web-based programs (42). In the study from Polzien et al. (43), *n* = 57 people participated in a 12-week weight loss program. The modules included individualized sessions addressing dietary and exercise modifications. Additionally, two groups received a web-based program to monitor eating habits and a wearable device monitoring energy expenditure. For both online groups, these features were provided either continuously or intermittently (during weeks 1, 5, and 9) during the intervention (43). The findings from this research group demonstrated that both online groups significantly decreased body weight, but with significantly more pronounced effects in the group with a continuous application of the online features (43).

Besides measures of weight loss, evidence strongly suggests that hypertension plays a key role in developing cardiovascular events (44). Thus, an indirect association between high blood pressure and COVID-19 outcomes can be assumed. The findings of the present study revealed that systolic blood pressure decreased by 5.1% and 1.7% in the intervention and control group, respectively, during the 12-week intervention phase.

As an important feature of cardiovascular diseases and hypertension, endothelial dysfunction is frequently discussed due to its relation to hypertension in pathophysiological mechanisms (45). Recent reports have impressively indicated that COVID-19 might be associated with endothelial damage and inflammation (46). In the present study, endothelial function was assessed by means of flow-mediated dilatation, and the results showed no significant differences over time in any of the study groups. This is not surprising since endothelial function is influenced by multiple moderators including visceral adiposity (47), smoking (47), caffeine use (48), and others (49).

All the presented lifestyle-related cardiometabolic risk factors together led to a significant decrease in COVIDAge by 4.2% in the intervention group but only 1.3% in the control group.

The present study is not without limitations. First, it needs to be mentioned that in this study, only participants of the intervention group with a program adherence of > 50% were included in the analyses. This approach was implemented since the primary aim of the present study was to investigate the extent to which regular attendance in a web-based weight loss program influences COVIDAge and lifestyle-related cardiometabolic risk factors. Therefore, participants with irregular attendance and thus adherence rates equal to or less than 50% were not included in the analyses. Moreover, we are aware that data from web-based interventions rely on self-reported data and might be biased by social desirability or errors in over- and underreporting. Nevertheless, web-based health programs are an effective tool for providing personalized health coaching and mimic face-to-face sessions in a time and cost-efficient way (50).

In conclusion, this study demonstrated that COVIDAge, a measure for the vulnerability of severe COVID-19 outcomes, can be modified by regular attendance in an interactive web-based weight loss program for adults with overweight and obesity. Additionally, significant decreases in lifestyle-related cardiometabolic risk factors, namely body weight, BMI, waist circumference, and systolic blood pressure, were observed, comparable to previously reported changes following face-to-face interventions (51). Further research should support these findings.

## Data Availability Statement

The datasets presented in this article are not readily available because the study protocol states that the study findings will only be released to the scientific community and the trial sponsor as results in aggregated form. All results supporting the conclusions of the manuscript are included in the manuscript. No other data will be shared with the scientific community or third parties. Requests to access the datasets should be directed to judith.brame@sport.uni-freiburg.de.

## Ethics Statement

The studies involving human participants were reviewed and approved by the Ethics Committee of the University of Freiburg. The patients/participants provided their written informed consent to participate in this study.

## Author Contributions

JB and DK designed the study. JB was responsible for data acquisition. JB, CC, and NB analyzed the data. JB, CC, NB, MB, AG, and DK interpreted the data and wrote the manuscript. All authors contributed to manuscript revision and finally read and approved the submitted version.

## Funding

This study was funded by Techniker Krankenkasse (Hamburg, Germany). The funder was not involved in the study design, collection, analysis, interpretation of data, the writing of this article, or the decision to submit it for publication The article processing charge was funded by the Baden-Wrttemberg Ministry of Science, Research and Art and the University of Freiburg in the funding program Open Access Publishing.

## Conflict of Interest

JB and DK report funding from Techniker Krankenkasse for the study design, implementation, and evaluation. The remaining authors declare that the research was conducted in the absence of any commercial or financial relationships that could be construed as a potential conflict of interest.

## Publisher's Note

All claims expressed in this article are solely those of the authors and do not necessarily represent those of their affiliated organizations, or those of the publisher, the editors and the reviewers. Any product that may be evaluated in this article, or claim that may be made by its manufacturer, is not guaranteed or endorsed by the publisher.

## Abbreviations

COVID-19: coronavirus disease 2019
BMI: body mass index
ONLINE: intervention group
CON: control group.
